# Decreased miR-155-5p, miR-15a, and miR-186 Expression in Gastric Cancer Is Associated with Advanced Tumor Grade and Metastasis 

**DOI:** 10.29252/.23.5.338

**Published:** 2019-09

**Authors:** Ali Zare, Behnam Alipoor, Mir Davood Omrani, Mohammad Reza Zali, Nasser Malekpour Alamdari, Hamid Ghaedi

**Affiliations:** 1 Department of Medical Genetics, School of Medicine, Shahid Beheshti University of Medical Sciences, Tehran, Iran;; 2 Department of Laboratory Sciences, Faculty of Paramedicine, Yasuj University of Medical Sciences, Yasuj, Iran;; 3 Urogenital Stem Cell Research, Shahid Beheshti University of Medical Sciences, Tehran, Iran;; 4 Gastroenterology and Liver Diseases Research Center, Research Institute for Gastroenterology and Liver Diseases, Shahid Beheshti University of Medical Sciences, Tehran, Iran;; 5 Department of General Surgery, Clinical Research and Development Unit at Modarres Hospital, Shahid Beheshti University of Medical Sciences, Tehran, Iran

**Keywords:** Dysplasia, Gastric cancer, MicroRNAs, MicroRNA stomach cancer

## Abstract

**Background:**

Gastric cancer (GC) is one of the most prevalent cancers with a high rate of mortality in the world. In recent years, microRNAs (miRNAs) have been proposed to be involved in GC development. In this study, we aimed at investigating differential expression level of miR-155-5p, miR-15a, miR-15b, and miR-186 in GC.

**Methods:**

For this research, we used qPCR to investigate miR-15b, miR-155, miR-15a, and miR-186 expression levels in a total of 29 normal gastric tissue, 45 gastric dysplasia, and 39 GC samples.

**Results:**

We showed significant down-regulation of miR-155-5p (*p* = 0.0018), miR-15a (*p* = 0.0159), and miR-186 (*p *= 0.0005) expression in GC tissue.

**Conclusion:**

This study provides evidence for deregulated expression of miR155-5p, miR-186, and miR-15a in GC and is providing new insights into the potential implication of these miRNAs in the pathogenesis of GC.

## INTRODUCTION

Gastric cancer (GC) is the fourth most prevalent cancer and the second leading cause of cancer-related mortality in the world. It is estimated that one million new cases are diagnosed with GC each year^[^^1^^,^^2^^]^. In spite of the North America, Japan, and Western Europe, the incidence of GC has been rising over the past 30 years in Iran, particularly northern and northwestern of Iran are high risk regions for GC^[^^3^^]^. Despite a marked decline in the incidence of GC, it remains a significant public health problem in developing countries^[^^4^^]^. Regardless of significant advances in diagnostic and therapeutic procedures, the five-year survival rate of GC remains very low. This is mainly because the majority of GC cases are diagnosed at advanced stages^[^^5^^]^. Thus, the understanding of molecular mechanisms underlying GC is urgently needed for the identifications of novel targets for accurate and effective diagnosis and treatment of the disease. A growing body of evidence suggests that microRNAs (miRNAs) are involved in major tumorigenesis pathways, and also dysregulation of miRNAs occurs virtually in all examined tumor types^[^^6^^,^^7^^]^.

MiRNAs are a class of small non-coding RNAs that modulate the expression of a wide variety of target mRNAs by inducing either translational inhibition or mRNA degradation^[^^8^^]^. Tissue miRNA expression profiles can effectively discriminate a wide range of disorders, including human malignancies. To date, several miRNA expression profiling studies have revealed a number of miRNAs as the potential therapeutic targets and diagnostic markers in cancers including GC^[^^9^^-^^11^^]^. However, a limited number of studies have investigated the roles of miRNAs in the determination of malignant transition from precancerous lesion such as gastric dysplasia (GD) to advanced GC^[^^12^^,^^13^^]^. In our previous report, we published a list of bioinformatically ranked miRNAs, which believed to play crucial roles in GC development based on the cancer genome atlas expression data^[^^13^^]^. Following the prioritization, we evaluated miR-335, miR-124, miR-218, and miR-484 expression and showed significant down-regulation of these miRNAs in both GD and GC tissues.In the present research, we explored the expression levels of four miRNAs, including miR-15b, miR-155, miR-15a, and miR-186 in normal gastric (NG), GD, and GC tissues. 

## MATERIALS AND METHODS

**Tissue samples**

We obtained a total of 113 formalin-fixed paraffin-embedded (FFPE) tissue blocks (29 NG, 45 GD, and 39 GC samples) from the Tissue Bank of the Gastroenterology and Liver Disease Research Center (Tehran, Iran). The study protocol was in complete compliance with the Helsinki declaration and approved by the Shahid Beheshti University of Medical Science Ethical Committee (SBMU.1396.583). Clinical and histopathological features of samples, including gender, age, stage of cancer, metastasis to lymph nodes, and *Helicobacter Pylori* infection were obtained from the medical records. The stool antigen test was used for *H. pylori* infection detection. The histology of samples was determined by a clear pathological report. The patients received neoadjuvant chemotherapy or radiotherapy were excluded from the study.

**RNA extraction**

Four to five 10-μm thick sections of FFPE tissue were used for total RNA extraction. In the case of GD and GC samples, only part of the sections with desired tissue type was included for further processing. To remove paraffin from FFPE tissue, the deparaffinization solution (Qiagen, Hilden, Germany) was used. For RNA extraction, we used the Qiagen miRNeasy FFPE kit (Qiagen, Hilden, Germany) according to the manufacturer’s protocol. Total RNA purity and concentration were determined by measuring the ratio of the absorbance at 260 and 280 nm using a Nano Drop™ 2000c Spectrophotometer (Thermo Fisher Scientific, USA). The extracted total RNA was stored at -70 °C until use.

**MiRNA quantitation**

Briefly, a poly-A tail was added to total RNAs (500 ng) using Poly-A polymerase (New England Biolabs, UK) at 37 °C for 30 min according to manufacturer’s protocol. For cDNA synthesis, we used PrimeScript™ 1st strand cDNA Synthesis Kit (TaKaRa, Japan) and an RT adaptor primer (Table 1). Real-time PCR was performed with SYBR Premix Ex TaqTM II (TaKaRa, Japan). The sequences of the qPCR primers for U6 and candidate miRNA are listed in Table 1. The PCR was done in duplicate according to the standard program on Rotor-Gene Q instrument (Qiagen, Hilden, Germany) using the following conditions: 95 °C for 10 seconds, followed by 30 cycles at 95 °C for 10 s and 60°C for 15 s, and finally a dissociation curve step. PCR efficiency was evaluated by LinRegPCR software (http://www.hartfaalcentrum.nl/; subject: LinRegPCR), and difference of the expression level for candidate miRNAs was calculated using the 2^- ΔΔCT^ method. 

**Table 1:** The sequence of primers used in this study

**Transcript**	**Sequence (5'3')**
RT primer	GCGAGCACAGAATTAATACGACTCACTATAGGTTTTTTTTTTTTTTVN
miR-15b	GGGTAGCAGCACATCATGGTTTA
miR-155-5p	GGGTTAATGCTAATCGTGATAGGG
miR-15a	GGGTAGCAGCACATAATGGTTTG
miR-186	GGGCAAAGAATTCTCCTTTTGGG
U6	CGCAAGGATGACACGCAAATTC
Universal	GCGAGCACAGAATTAATACGACTC

**Table 2:** Demographic and histopathological characteristics of the study subjects

**Parameter**	**NG (n = 29)**	**GD (n = 45)**	**GC (n= 39)**	***p*** **value**
Age (y)	50.58 ± 16.52	55.24 ± 14.47	53.15 ± 13.61	0.58
Sex (male/female)	17/12	23/22	21/18	0.81
*H.* * pylori* (positive/negative)	6/23	10/35	22/17	0.001

Age data are represented as mean ± SD.

**Pathway enrichment**

A list of experimentally validated target genes for candidate miRNAs was obtained from miRTarbase v. 4.5^[^^14^^]^ and StarBase v. 2.0^[^^15^^]^ databases. We used the Databases for annotation, visualization, and integrated discovery (DAVID) tool^[^^16^^]^ for pathway enrichment analysis. 

**Statistical analysis**

All statistical analyses were performed by SPSS Statistical Software Package (version 18.0). The expression levels of the miRNAs between two groups were evaluated using student’s 𝑡-test for the normally distributed data or Mann-Whitney U test for the nonparametric data. ANOVA and Kruskal-Walis tests were used for the comparisons of miRNA expression levels between more than three groups for the normally distributed and for the nonparametric data, respectively. *p* values less than 0.05 were considered to be statistically significant. 

## RESULTS

**Study population**

Demographic and histopathological characteristics of the study subjects (29 NG, 45 GD, and 39 GC samples) are presented in the Table 2. The study groups were matched for age and sex (*p* = 0.58 and *p* = 0.81, respectively). We observed significant differences between the groups regarding to the *H. pylori* infection (*p* = 0.001).

**M** **iRNA** **expression signature**

**Fig. 1:**
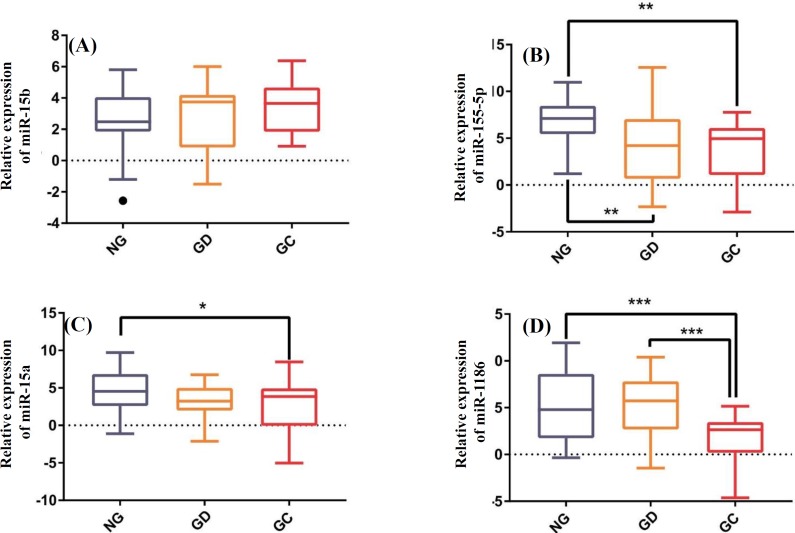
Relative expression of miR-15b (A), miR-155-5p (B), miR-15a (C), and miR-186 (D) in gastric cancer (GC), gastric dysplasia (GD), and normal gastric (NG) tissues. *, **, and *** indicate *p* ≤ 0.05, *p* ≤ 0.01, and *p* ≤ 0.001, respectively.

The expression levels of miR-15b, miR-155-5p, miR-15a, and miR-186 were compared between NG, GD, and GC tissues. Our analysis revealed that the expression levels of miR-155-5p (*p* = 0.0018), miR-15a (*p* = 0.0159), and miR-186 (*p *= 0.0005) showed a significant down-regulation in GC tissue; however, we did not observe a significant difference for miR-15b expression between GC and NG groups (*p* = 0.3491; Fig. 1). In addition, we found that the miR-155-5p expression level significantly decreased in GD compared to NG (*p *= 0.0015) tissues. Moreover, the results showed that miR-186 expression in GC was significantly lower than that in GD (*p *= 0.0002).

**M** **iRNA** **expression and clinicopathological characteristics**

As shown in the Table 3, GC patients with lymph node metastasis had a lower expression of miR-155-5p, miR-15a, and miR-186 expression (*p *< 0.05). Moreover, patients at the advanced stages (III, IV) of GC had a much lower expression of miR-155-5p, miR-15a, and miR-186 than patients at early stages (I, II; *p* < 0.05). Further, the evaluation of the relationship between the expression of the candidate miRNAs and *H. pylori* infection revealed that the miR-155 expression level significantly decreased in the *H*.* pylori*-positive cancer tissues compared to the infection-free cancer tissues (*p* = 0.01). 

**Pathways enrichment analysis**

To further investigate the possible implication of miR-155-5p, miR-15a, and miR-186 in GC development, we conducted a pathway enrichment analysis on the experimentally validated target genes of these miRNAs by the DAVID tool^[^^16^^]^. Several cancer related pathways (e.g. Pathways in cancer_Homo sapiens_hsa05200, Chronic myeloid leukemia_Homo sapiens_hsa05220, and Colorectal cancer_Homo sapiens_hsa05210) and cell signaling pathways (e.g. Cell cycle_Homo sapiens_hsa04110, Ras signaling pathway_Homo sapiens_hsa04014, MAPK signaling pathway_Homo sapiens_hsa04010, and Hippo signaling pathway_Homo sapiens_hsa04390) were enriched by target genes of miR-155-5p, miR-15a, and miR-186. 

## DISCUSSION

Nowadays, the majority of GC cases are diagnosed at advanced stages, and consequently, therapeutic options are very limited and not effective. Therefore, the advent of novel diagnostic methods and appropriate biomarkers is a prerequisite for early detection and treatment of GC^[^^5^^]^. Understanding molecular changes in precancerous and tumor tissues provide an opportunity to obtain deeper insights into carcinogenesis.

In this study, we investigated the expression of miR-15b, miR-155-5p, miR-15a, and miR-186 in GC compared to GD and NG tissues. Our results revealed that the expression levels of miR-155-5p, miR-15a, and miR-186 were significantly down-regulated in GC; however, we did not observe a significant difference for miR-15b expression. 

The miR-155 is among the most well-studied miRNAs for its roles in modulating the immune response and importantly for its role in a range of human cancer^[^^17^^,^^18^^]^. There are significant inconsistencies regarding the role of miR-155 in tumorigenesis in the literatures. On the one hand, there is considerable evidence suggesting that the miR-155 may act as oncomiR^[^^17^^,^^19^^,^^20^^]^. On the other hand, newly emerged shreds of evidence, especially in the field of GC, strongly support a tumor suppressor role for this miRNA^[^^21^^]^. In this study, we found significant down-regulation of miR-155 in GC. Similarly, our data revealed a marked down-regulation for this miRNA in GD as compared to NG. This observation implies that deregulation of miR-155 may be an early molecular aberration in GC development. Moreover, our results showed that the expression level of miR-155 reduced in *H. pylori*-positive GC cases as compared to infection-free tumor tissues. It has widely been acknowledged that miR-155-5p is involved in inflammatory pathways implicated in *H. pylori-*induced gastritis and GC^[^^22^^,^^23^^]^. Notable findings have shown that miRNAs are deregulated in early stages of gastric carcinogenesis like *H. pylori*-induced gastritis and premalignant stages such as GD^[^^22^^]^. Further, we found that the expression of miR-155 is significantly repressed in tumors at advanced stages and also in tumors invaded lymph nodes. These data more support the tumor suppressor role of miR-155 in the GC development.

Our results revealed significant down-regulation of miR-186 in GC compared to GD and NG samples. Since this repression is only evident in GC and not GD, it is conceivable that miR-186 dysregulation is a late molecular change in GC development. It has become clear that miR-186 acts as a tumor suppressor in several human cancers and also could modulate sensitivity to chemotherapeutics^[^^24^^]^. Researchers have demonstrated that miR-186 may affect several neoplastic characteristics of GC cells, including proliferation, invasion, and migration^[^^25^^]^. Also, Huang *et al*.^[^^26^^]^ have demonstrated that miR-186 is signiﬁcantly repressed in highly differentiated gastric adenocarcinoma tissues. As another line of evidence that supports a tumor suppressor role for miR-186, we found significant down-regulation of this miRNA in GC samples with lymph node metastasis. 

**Table 3:** The microRNAs expression levels (medians of ΔCt and interquartile range) according to the clinicopathological characteristics of the study subjects

**Characteristics**	**N**	**miR-155-5p**	***p value***	**miR-15a**	***p*** ***value***	**miR-186**	***p*** ***value***
Stage I,II III,IV	16 23	5.92(2.24-8.19) 7.70(6.31-9.93)	0.03	3.78(2.48-4.73)	0.02	4.71(2.32-5.91)	0.00
				5.81(4.33-7.02)		8.42(4.47-10.41)	
LNM Positive Negative	18 21	7.10(6.19-9.12) 5.14(1.39-7.52)	0.02	5.54(3.73-6.85)	0.01	4.14(1.99-5.94)	0.04
				3.99(0.30-4.74)		3.84(1.84-5.59)	
*H. pylori *(GC) Positive Negative	22 17	6.12(4.36-8.27) 5.00(2.08-7.00)	0.01	4.45(2.30-6.12) 4.52(2.13-6.12)	0.83	4.45(1.22-7.90)	0.93
						4.61(2.68-6.61)	
*H. pylori* (healthy controls) Positive Negative	16 58	5.68(3.66-7.38) 4.30(1.87-6.20)	0.06	4.74(3.42-6.11) 4.52(2.11-6.76)	0.56	4.90(2.10-7.46) 5.39(3.33-7.00)	0.91

*p* values were obtained by Mann–Whitney U test. LNM, lymph node metastasis;

Similar to a previous report, our analysis showed that the expression level of miR-15a was significantly lower in GC samples compared to NG subjects^[^^27^^]^. MiR-15a/16-1 gene cluster locates in chromosome 13 of the human genome and mainly acts as a tumor suppressor by promoting apoptosis and inhibiting cell proliferation^[^^21^^]^. Furthermore, GC patients with lymph node metastasis and also patients with advanced tumor grade had a lower expression of miR-15a. It has been reported that patients with higher expression levels of miR-15a has better outcomes compared with those who do not have high levels of this miRNAs^[^^21^^]^. 

Although the GC group sample size in the present study was not large enough; however, we showed a significant correlation between miR-155, miR-15a, and miR-186 expression levels and tumor stage or lymph node metastasis. Further experimental studies with a large sample size are needed to verify the robustness of the obtained results.

Finally, our analysis showed that the pathways enriched by miR-155, miR-15a, and miR-186 were mainly centralized in pathways related to tumorigenesis (Colorectal cancer_Homo sapiens_ hsa05210, Chronic myeloid leukemia_Homo sapiens_ hsa05220, Viral carcinogenesis_Homo sapiens_ hsa05203, Pathways in cancer_Homo sapiens_ hsa05200), cell cycle (Cell cycle_Homo sapiens_ hsa04110, Signaling pathways regulating pluripotency of stem cells_Homo sapiens_hsa04550), and cell signaling (Oxidative phosphorylation_Homo sapiens_ hsa00190, Rap1 signaling pathway_Homo sapiens_ hsa04015, Ras signaling pathway_Homo sapiens_ hsa04014, Hippo signaling pathway_Homo sapiens_ hsa04390, MAPK signaling pathway_Homo sapiens_ hsa04010). Such data more support the implication of these miRNAs in GC development. 

In summary, the results of this study support the deregulated expression of miR155-5p, miR-186, and miR-15a in GC and provide new insights into the potential implication of these miRNAs in the pathogenesis of GC. However, a comprehensive study should be carried out on the target genes of these miRNAs to clarify the possible role(s) of these genes in GC development.
